# Neuromyelitis Optica Spectrum Disorder with Sick Sinus Syndrome: Two Cases and a Literature Review

**DOI:** 10.3390/healthcare11212810

**Published:** 2023-10-24

**Authors:** Huiting Lin, Xinyi Duan, Lina Li, Jinhao Ye, Haibing Xiao

**Affiliations:** 1 Department of Neurology, Neuromedicine Center, The University of Hong Kong-Shenzhen Hospital, Shenzhen 518000, China; linht@hku-szh.org (H.L.); duanxy@hku-szh.org (X.D.); liln@hku-szh.org (L.L.); yejh@hku-szh.org (J.Y.); 2 Shenzhen University Medical School, Shenzhen University, Shenzhen 518000, China

**Keywords:** neuromyelitis optica spectrum disorders, NMOSD, sick sinus syndrome, SSS, medulla oblongata, area postrema syndrome

## Abstract

Background and objective: Neuromyelitis optica spectrum disorder (NMOSD) is a rare immune-mediated demyelinating disease of the central nervous system (CNS). There is a lack of reports of sick sinus syndrome (SSS) associated with NMOSD; thus, we hereby report two cases of patients with NMOSD who developed SSS. Cases presentation: The patients were both male and presented with area postrema syndrome. Brain MRI showed lesions in the dorsal part of their medulla oblongata. They were diagnosed with NMOSD when aquaporin-4 antibodies were found in their serum. Slow heart rates and several episodes of syncope were also observed in case 1 during hospitalization, while Holter monitoring showed sinus pauses (10–11 s) and SSS was diagnosed. A pacemaker was fitted. Case 2 had a respiratory arrest followed by a subsequent cardiac arrest. He was successfully resuscitated with epinephrine injection and cardiopulmonary resuscitation. Through immunotherapy, their neurological functions became stable and heart rate and blood pressure returned to the baseline. Conclusions: Since sick sinus syndrome is a life-threatening complication, serious heart arrhythmias should be considered as a potential result of area postrema syndrome associated with NMOSD.

## 1. Introduction

Neuromyelitis optica (NMO) is an inflammatory CNS disorder that is associated with anti-aquaporin-4 immunoglobulin G (AQP4-IgG). After its relationships with brain lesions in the area postrema, diencephalon, cerebrum, and other brainstem areas were recognized, its broad continuum of clinical presentations has given rise to the concept of NMO spectrum disorders (NMOSDs) [1]. Sick sinus syndrome is a type of disorder caused by impaired pacemaker functions of the sinoatrial node and its adjacent tissues, resulting in a variety of arrhythmias and other clinical symptoms (such as syncope and sudden cardiac death). Most patients present with other complications such as coronary heart disease, cardiomyopathy, or other heart diseases, and symptoms develop between the ages of 60 and 70 [2]. As a life-threatening complication, SSS is rarely documented in NMOSD patients. This study therefore reports two cases of patients with NMOSD who developed SSS, while a total of eight cases with both NMO and SSS have been reviewed. Based on these observations, we consider that SSS may be related to medulla oblongata lesions that affect cardiac function and give rise to serious arrhythmias and hemodynamic disorders. In addition, we have also noticed that this potential mechanism is disproportionately more likely to occur in male patients.

## 2. Methods

Two cases of anti-AQP4 antibody-positive NMOSD in patients with a history of SSS were diagnosed at the University of Hong Kong-Shenzhen Hospital between December 2021 and April 2022. The patients were informed that deidentified data concerning the case would be submitted for publication, and consent was provided.

The diagnostic criteria for NMOSD with AQP4-IgG refer to the revised consensus NMOSD clinical diagnostic criteria proposed by the International Panel for NMO Diagnosis (IPND) in 2015 [3].

Diagnostic criteria for NMOSD with AQP4-IgG:

1. At least one core clinical characteristic.
2. Positive test for AQP4-IgG using the best available detection method (a cell-based assay is strongly recommended).
3. Exclusion of alternative diagnoses.

Core clinical characteristics:

1. Optic neuritis;
2. Acute myelitis;
3. Area postrema syndrome—an episode of otherwise unexplained hiccups or nausea and vomiting;
4. Acute brain stem syndrome;
5. Symptomatic narcolepsy or acute diencephalic clinical syndrome with NMOSD-typical diencephalic MRI lesions;
6. Symptomatic cerebral syndrome with NMOSD-typical brain lesions.

## 3. Results

Two cases were diagnosed at the University of Hong Kong-Shenzhen Hospital.

### 3.1. Case 1

**Figure 1:**
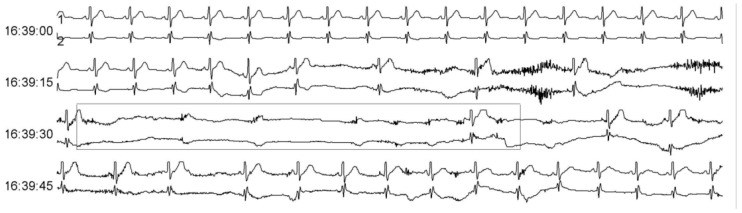
Holter monitoring showing sinus arrest with the longest RR interval at about 10 s.

A 45-year-old man presented with acute onset of hiccups and vomiting for nearly two weeks prior to admission. Gastrointestinal endoscopy revealed chronic superficial gastritis, and infection-related tests were negative. The cause of the hiccups remained unclear, while metoclopramide, haloperidol, and chlorpromazine did not relieve the hiccups and vomiting. He had no past medical history, and the results of his vital signs included a blood pressure of 91/56 mmHg, a pulse of 48 bpm, and a normal sinus rhythm. No other abnormal pulmonary, cardiac, or abdominal findings were confirmed, while the results of his neurological examination were normal, except for bilaterally absent deep tendon reflexes. During hospitalization, he had slow heart rates that ranged from 30 to 60 bpm with several episodes of syncope. Moreover, ECG monitoring indicated the occurrence of sinus pauses with a duration of about 10 s, while Holter monitoring showed recurring cardiac arrest with the longest RR interval of 11 s (Figure 1). He was subsequently diagnosed with SSS, before temporary pacemaker implantation surgery was performed.

**Figure 2:**
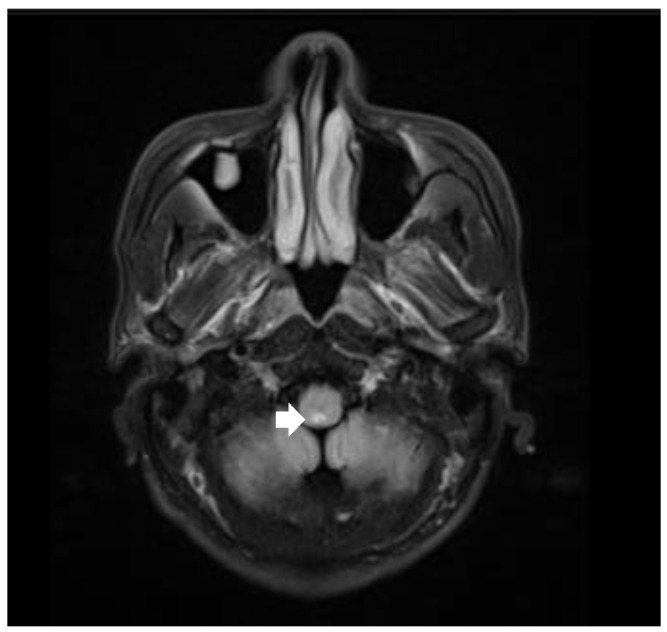
Brain MRI revealed bilateral high-intensity lesions in the dorsal part of the medulla oblongata on axial fluid attenuated inversion recovery (FLAIR) sequence.

As for laboratory examinations, positive serological results included elevated anti-aquaporin-4 antibody and anti-SS-A antibody (+++) levels. CSF examination showed normal cell count and protein values. Cardiac ultrasound, EEG, and abdominal imaging with contrast-enhanced CT did not confirm any abnormalities. However, a high-intensity lesion was found in the dorsal part of the medulla oblongata in brain MRI (Figure 2). The patient was diagnosed with NMOSD accompanied by SSS and Sjögren syndrome. His SSS was cured, followed by the disappearance of the hiccups and vomiting, after he was treated with intravenous IVIG (0.4 g/kg × d × 5 days) and methylprednisolone (1000 mg/d × 5 days). In addition, both his heart rate and blood pressure stabilized at around 70 bpm and 100/60 mmHg, respectively, after the temporary pacemaker was removed. He had no neurological or cardiac sequelae after the initiation of immunotherapy. After discharge, the patient gradually reduced and then stopped his intake of oral steroids and started using Rituximab as a disease-modifying therapy. The patient’s condition remained stable with no relapses.

### 3.2. Case 2

A 26-year-old male patient was diagnosed as having NMOSD before the present study after presenting with vomiting, hiccups, and double vision 2 years before. An AQP4 antibody test was positive, and diplopia lingered on after methylprednisolone therapy. He developed limb weakness six months prior to this study, and was then treated with methylprednisolone therapy again, though he did not take the immunosuppressants regularly. After he was readmitted to our hospital with the sudden onset of slurred speech, dysphagia, hiccups, diplopia, and limb weakness ten days prior to readmission, neurological evaluation showed the existence of left facial nerve palsy, an eye movement disorder, and nystagmus. Besides the obvious hiccups and dysphagia, manual muscle testing on his lower extremities showed a grade 4 result. Tendon reflexes were diminished, though no pathological reflexes were observed. Pain sensations were impaired in the left sides of his face and his body.

**Figure 3:**
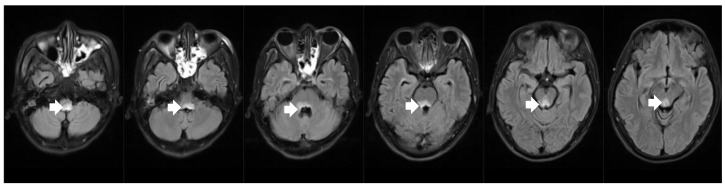
Brain MRI revealed bilateral high-intensity lesions in the dorsal part of the medulla oblongata, periependymal surfaces of the pons and dorsal midbrain on axial fluid attenuated inversion recovery (FLAIR) sequence.

Brain MRI showed new lesions in the dorsal part of the medulla oblongata, pons, and mesencephalon (Figure 3). A serum anti-aquaporin 4 antibody test was positive. Based on typical brainstem syndrome manifestations, the presence of periependymal brain stem lesions in MRI, and the positive AQP4 antibody test, this patient was definitively diagnosed with NMOSD. After readmission, the patient was referred to the ICU due to aspiration pneumonia caused by dysphagia, as well as the recurring hiccups and vomiting. After strong anti-infection therapy, the patient’s body temperature returned to normal, and chest CT showed inflammation resorption and improvements in inflammatory factors. Nevertheless, he had a respiratory arrest followed by a subsequent cardiac arrest. He was successfully resuscitated with epinephrine injection and cardiopulmonary resuscitation. Considering the patient’s pulmonary infection was under control, and his symptoms were not consistent with the presentation of septic shock, we attributed the cause of the slow heart rate and low blood pressure to his NMOSD. The patient was treated with one course of intravenous methylprednisolone, one course of therapeutic plasma exchange, and one course of IVIG before his blood pressure and heart rate were stable within the normal range. He was then transferred to another hospital for rehabilitation. After discharge, the patient chose oral steroids and azathioprine as a disease-modifying therapy.

## 4. Discussion

We report two patients with NMOSD of the dorsal medulla oblongata who presented with area postrema syndrome and potentially fatal bradyarrhythmia or cardiac arrest due to diagnosed SSS. The possibility that NMOSD may also induce SSS has not been shown much consideration. Only a few patients with NMOSD who presented with SSS due to medullary lesions have been reported. We have reviewed all previously reported NMOSD cases with SSS in a total of eight articles after searching PubMed and Medline [4,5,6,7,8,9,10,11]. Table 1 summarizes the eight previous reports of NMOSD patients with SSS and our report of two patients (patients No. 9 and No. 10). In this comparison, we aimed to discover common characteristics between these patients.

As we know, NMOSD has a prevalence of approximately 0.52–4.4 per 100,000 people, with a significantly higher predominance in females. The proportion of female AQP4-IgG-seropositive individuals increases exponentially after the age of 50 [12,13,14]. However, we have also noticed that sick sinus syndrome is more likely to occur in male patients (male vs. female ratio of 6:4). Considering that the ratio of NMOSD prevalence in men and women is 1:9, and that of SSS with NMO is 6:4, a gender difference in SSS morbidity seems to exist, though with an unclear mechanism. Therefore, gender is likely to be a predisposing factor for the complication of SSS with NMO, with more cases and studies required to verify this.

The cause of SSS in NMOSD remains unknown. Sick sinus syndrome is a slow heart rhythm disorder caused by an abnormal sinus node function, mainly occurring in elderly people or patients with organic heart disease. In these cases, the correlation between age and bradyarrhythmia is not significant (the age ranges from 16 to 78 years old). Similar to our cases, most of the reported cases in the literature were not linked to underlying heart diseases before illness onset, and there were no positive findings from cardiac-related examinations after onset. Moreover, myocarditis, which might be due to the expression of various aquaporins by the myocardium, including AQP4, has been previously reported in an NMOSD patient, which indicates that severe myocarditis may result in arrhythmia [15,16]. However, this possibility has been excluded by our observations of normal myocardial enzyme levels and cardiac ultrasound results in our patients. It is also worth noting that all patients had lesions in the dorsal or lower part of their medulla oblongata and developed area postrema syndrome. Slow arrhythmia occurred with neurological symptoms, and in some patients, it even appeared before the onset of neurological symptoms. After immunotherapy, most patients were weaned off the temporary pacing support, which indicates that SSS and area postrema syndrome are caused by parallel lesions in the medulla oblongata. SSS caused by NMOSD is reversible, and the patient’s pacemaker can be removed when the neuroinflammation is controlled, which is in contrast to general patients, who require a permanent pacemaker.

Previous studies have identified the chemoreceptors that trigger vomiting in the dorsal gray matter of the medulla oblongata, which is defined as the nucleus of the solitary tract (NST). The NST plays a key role in the vomiting reflex via stimuli from the area postrema. Thus, area postrema syndrome is characterized primarily by persistent and unexplained hiccups or vomiting. Sick sinus syndrome and syncope, however, are not frequently observed in NMOSD. To the best of our knowledge, the NST and other related brainstem nuclei all play important roles in cardiovascular function regulation. The parasympathetic (vagal) baroreflex is processed at the nucleus ambiguus (NA) and the dorsal motor nucleus of the vagus (DMV), acting on the sinoatrial node of the heart. The NST, meanwhile, receives input through sympathetic afferent fibers from arterial baro- and chemo-receptors and transmits stimuli via efferent fibers to the rostral ventrolateral medulla (RVLM). The sympathetic outflow moves to the interomediolateral cell column of the spinal cord and regulates its effector organs (heart and blood vessels). Parasympathetic and sympathetic neural pathways are responsible for heart rate regulation, cardiac output, and blood pressure [17,18]. It is therefore evident that inflammation and its subsequent damage to the NST, as well as to its numerous projection fibers to the RVLM, the NA, and the DMV, can affect cardiac function and result in serious arrhythmias and hemodynamic disorders. In a previous report, a patient with NMOSD exhibiting intractable hiccups and orthostatic hypotension as initial symptoms was described, presenting with a lesion from the medulla oblongata to the upper cervical cord visible on MRI [3]. Both of our patients had lesions in the dorsal part of the medulla oblongata, but did not have orthostatic hypotension. Thus, APS may have a diverse range of symptom severity. The reasons for the difference in clinical symptoms are still unclear, and more case reports and literature support are needed, which is also a shortcoming of this article.

## 5. Conclusions

The medullary lesions in NMOSD patients may change the balance between sympathetic and vagal activity, influencing their base heart rate and blood pressure. Clinicians should be aware of the possibility that medullary lesions in NMOSD patients can cause SSS, especially in male patients. It is imperative to perform long-term ECG monitoring on these patients to identify potentially fatal bradyarrhythmia as early as possible.

## Figures and Tables

**Table 1:** Patients of NMOSD with arrhythmia.

Patient No.	Age	Sex	Arrhythmia	Neurological Symptoms	MRI Findings	History of Heart Diseases	Treatment for NMOSD
1 [4]	78	M	Cardiac arrest	Syncope, orthostatic hypotension, APS, lower limb weakness	Medulla oblongata and cervical cord	No	IVMP, IVIG
2 [5]	21	F	Cardiac arrest	APS	Medulla oblongata	No	IVMP, RTX
3 [6]	77	M	Sinus arrest	APS	Medulla oblongata	CHD	Oral prednisolone
4 [7]	61	F	Sinus arrest	Syncope, APS	Medulla oblongata	No	IVMP
5 [8]	42	M	Sinus arrest	Syncope, APS, left optic neuritis	Medulla oblongata and left optic nerve	No	IVMP
6 [9]	77	F	Sinus arrest	Syncope, APS, limb weakness	Medulla oblongata and cervical cord	No	IVMP, PE
7 [10]	16	M	Sinus bradycardia	APS	Medulla oblongata	No	IVMP
8 [11]	22	F	Sinus arrest	APS, syncope, diplopia and hypesthesia	Medulla oblongata and cervical cord	No	IVMP
9	45	M	Sinus arrest	Syncope, APS	Medulla oblongata	No	IVMP, IVIG
10	26	M	Cardiac arrest	APS, limbs weakness, dysphagia, diplopia	Medulla oblongata, pons and mesencephalon	No	IVMP, PE, IVIG

APS: area postrema syndrome; IVMP: intravenous methylprednisolone; IVIG: intravenous immunoglobulin; PE: plasma exchange; CHD: coronary heart disease.

## Data Availability

We present case reports of two patients, and to protect privacy and respect confidentiality, none of the raw data have been made available in any public repository. The original reports, laboratory studies, imaging studies, and outpatient clinic records were retained as per normal procedure within the medical records of our institution.

## Abbreviations:

NMOSD	Neuromyelitis optica spectrum disorder
CNS	central nervous system
SSS	sick sinus syndrome
MRI	magnetic resonance imaging
IVIG	intravenous immunoglobulin
ICU	intensive care unit
AQP4-IgG	anti-aquaporin-4 immunoglobulin
ECG	electrocardiograph
APS	area postrema syndrome
IVMP	intravenous methylprednisolone
PE	plasma exchange
CHD	coronary heart disease
NST	nucleus of the solitary tract
NA	nucleus ambiguus
DMV	dorsal motor nucleus of the vagus
RVLM	rostral ventrolateral medulla
FLAIR	fluid attenuated inversion recovery
